# Clinical characteristics and prognosis of peritoneal dialysis-associated peritonitis caused by non-fermenting Gram-negative bacteria: a single-center retrospective analysis

**DOI:** 10.1080/0886022X.2026.2679858

**Published:** 2026-06-24

**Authors:** Huiling Yan, Wenhui He, Dongrong Yu, Mengli Tong, Jun Ni, Jin Yu

**Affiliations:** Department of nephrology (Key laboratory of Kidney Disease Prevention and Control Technology), Hangzhou TCM Hospital affiliated to Zhejiang Chinese Medical university, Hangzhou, China

**Keywords:** Peritoneal dialysis, non-fermenting gram-negative bacteria, peritoneal dialysis-associated peritonitis, prognosis

## Abstract

This study compared the clinical characteristics and prognosis of peritoneal dialysis-associated peritonitis (PDAP) caused by non-fermenting Gram-negative bacteria (NF-GNB) versus fermenting Gram-negative bacteria (F-GNB). We analyzed 32 episodes of NF-GNB PDAP at our center from January 2010 to December 2024 and matched them 1:1 with F-GNB PDAP episodes by sex, age, and dialysis duration. The NF-GNB group had a higher prevalence of diabetes and a higher Charlson comorbidity index, but lower serum albumin, parathyroid hormone, neutrophil-to-lymphocyte ratio, calcium, and admission body temperature (all *p* < 0.05). The overall medical cure rate was lower in the NF-GNB group than in the F-GNB group (53.1% vs. 81.3%, *p* = 0.017). In the NF-GNB group, markedly elevated inflammatory markers (blood white blood cell count ≥ 10 × 10^9^/L, C-reactive protein ≥ 90 mg/L, or dialysate white blood cell count on day 3 ≥ 264/μL) or hypoalbuminemia (albumin < 30 g/L) might be associated with a higher risk of treatment failure. All-cause mortality during follow-up was higher in the NF-GNB group than in the F-GNB group (58.6% vs. 30.0%, *p* = 0.027), and lower survival was confirmed by Kaplan–Meier analysis (log-rank *χ*^2^ = 4.343, *p* = 0.037). NF-GNB infection (HR = 3.40, 95% CI: 1.05–11.00, *p* = 0.041) and lower *Kt/V* (HR = 12.54, 95% CI: 2.32–67.94, *p* = 0.003) might be associated with a higher risk of mortality. In conclusion, patients with NF-GNB PDAP have more comorbidities, poorer nutritional status, and worse clinical prognosis than those with F-GNB infection.

## 1. Introduction

Peritoneal dialysis (PD) is one of the important renal replacement therapies for patients with end-stage renal disease. However, peritoneal dialysis-associated peritonitis (PDAP), as a common and serious complication, often leads to technique failure and even patient mortality [1,2]. In recent years, the incidence of PDAP has declined, attributed to standardized peritoneal dialysis training and the widespread use of mupirocin [3]. Bacterial infections remain the most common cause of PDAP. Although Gram-positive bacteria (GPB) remain the predominant pathogens, the proportion of infections caused by Gram-negative bacteria (GNB) has been increasing annually, accounting for approximately 20–30% of cases [4,5]. Compared with GPB, PDAP caused by GNB is generally associated with lower remission rates, higher mortality, and poorer clinical outcomes [6–8].

Non-fermentative Gram-negative bacteria (NF-GNB) represent an important category of GNB. Unlike other GNB, they cannot ferment carbohydrates to produce acid only *via* oxidative pathways. As opportunistic pathogens that are widely distributed in both natural environments and healthcare settings, they are frequently implicated in respiratory, urinary tract, and skin infections. Among them, *Pseudomonas* and *Acinetobacter* are the predominant species. NF-GNB can produce a variety of virulence factors to facilitate adhesion, disrupt host cell membranes, and suppress macrophage responses; they are also capable of forming biofilms that enable survival under harsh environmental conditions; furthermore, they often exhibit high levels of antimicrobial resistance [9,10]. Of particular concern is their resistance to carbapenems, which poses a serious threat in hospital settings. Carbapenem-resistant *Acinetobacter baumannii* and *Pseudomonas aeruginosa* have been listed by the World Health Organization among the most critical priority pathogens posing the greatest threat to human health [11]. These intrinsic characteristics contribute to poor clinical prognosis in treating infections caused by NF-GNB.

NF-GNB PDAP is relatively uncommon, with only a few studies reporting on it. However, it represents an important group of pathogens responsible for severe peritonitis [3] and poses substantial challenges in clinical management. Therefore, this study aims to summarize the clinical characteristics of NF-GNB PDAP over the past 15 years at our center and evaluate its treatment outcomes.

## 2. Materials and methods

### 2.1. Study participants

This study retrospectively collected clinical data from patients undergoing continuous ambulatory peritoneal dialysis (CAPD) who developed PDAP at Hangzhou Hospital of Traditional Chinese Medicine Affiliated to Zhejiang Chinese Medical University between January 2010 and December 2024. The diagnosis of PDAP was based on the International Society for Peritoneal Dialysis (ISPD) guidelines [3], meeting at least two of the following three criteria: (1) presence of peritonitis-related clinical symptoms, such as abdominal pain and/or cloudy dialysis effluent; (2) dialysis white blood cell (WBC) count > 100/μL after a dwell time of over 2 h, with polymorphonuclear neutrophils accounting for > 50%; (3) positive microbial culture of the dialysis effluent. Based on the results of pathogen culture, patients with NF-GNB infection were assigned to the NF-GNB group. Using a 1:1 nearest-neighbor matching method without replacement, NF-GNB patients were propensity score matched with contemporaneous patients infected with fermentative Gram-negative bacteria (F-GNB) according to sex, age, and dialysis duration. The matching caliper was set to an absolute difference in propensity score not exceeding 0.02. After matching, the F-GNB group was obtained. The results showed that the standardized mean differences for all variables after matching were less than 0.1.

### 2.2. Data collection

All clinical data in this study were obtained from electronic medical records in the hospital information system. Collected information included patient demographics, clinical symptoms and signs, laboratory parameters, and prognosis. Demographics consisted of age, sex, dialysis duration, primary renal disease, Charlson comorbidity index (CCI), educational attainment, history of previous PDAP, and the urea clearance index (*Kt/V*). Clinical symptoms included abdominal pain, cloudy peritoneal dialysate, fever, nausea/vomiting, and diarrhea. Clinical signs mainly comprised body temperature, heart rate, and blood pressure at admission. Laboratory parameters included hematological indices and peritoneal dialysis fluid test results, with initial specimen testing completed within 24 h of admission. Hematological parameters included procalcitonin (PCT), C-reactive protein (CRP), white blood cells (WBC) count, neutrophils count, lymphocytes count, hemoglobin, parathyroid hormone (PTH), ferritin, albumin, high-density lipoprotein (HDL), low-density lipoprotein (LDL), lactate dehydrogenase (LDH), alkaline phosphatase (ALP), and electrolytes (potassium, calcium). Peritoneal dialysis fluid analysis included etiologic culture results and dialysate WBC count on day 1 and 3.

Definitions were as follows: body temperature > 37.5 °C was defined as fever; serum potassium < 3.5 mmol/L was defined as hypokalemia. The most recent *Kt/V* measurement within the three months prior to the onset of peritonitis was used as the basis for assessing dialysis adequacy, with a value of < 1.7 defined as inadequate dialysis. Comorbidity burden was classified into three categories based on the CCI [12]: low (2 points, as the baseline score for renal failure is 2), intermediate (3–4 points), and severe (≥ 5 points). The neutrophil-to-lymphocyte ratio (NLR) was calculated by dividing the peripheral blood neutrophil count by the lymphocyte count.

### 2.3. Research methods

#### 2.3.1. Treatment regimen

All patients with peritonitis received immediate empirical anti-infective therapy after adequate specimens were collected for microbial culture. The empirical regimen was required to cover both GPB and GNB. Specifically, GPB coverage was achieved with a first-generation cephalosporin or vancomycin, while GNB coverage was provided by a third-generation cephalosporin or an aminoglycoside. After definitive bacterial culture results were obtained, the anti-GPB therapy was discontinued, and the final antibiotic regimen was based on local pathogen antimicrobial susceptibility testing results and the patient’s clinical response. The standard treatment duration is two weeks. For patients with *Pseudomonas* infection or delayed clinical response, two sensitive antibiotics with different mechanisms of action are typically used in combination, and the treatment course is extended to three weeks. Catheter removal may be considered in patients presenting with any of the following: refractory peritonitis, or peritonitis complicated by refractory exit-site infection, or peritonitis complicated by tunnel infection. Concurrently, active pharmacological management and nutritional support were provided for comorbidities and complications such as diabetes mellitus, coronary heart disease, hypertension, renal anemia, and renal osteodystrophy.

#### 2.3.2. Prognostic assessment

Clinical prognosis was evaluated in accordance with the ISPD guideline criteria [3], and included both short-term and long-term prognosis. Short-term prognosis was analyzed at the episode level, and long-term prognosis at the patient level.

Short-term prognosis includes the following indicators: Relapsing: peritonitis episode that occurs within 4 weeks of completion of therapy of a prior episode with the same organism or one sterile (culture negative) episode; Recurrent: peritonitis episode that occurs within 4 weeks of completion of therapy of a prior episode but with a different organism; Peritonitis-associated hemodialysis (HD) transfer: transfer from PD to HD for any period of time as part of the treatment for a peritonitis episode; Peritonitis-associated death: referred to death occurring within 30 days of the peritonitis episode, or death during the hospitalization for peritonitis. Medical cure: referred to complete resolution of peritonitis together with none of the following complications: relapse/recurrent peritonitis, catheter removal, transfer to HD for ≥ 30 days or death. In this study, short-term prognosis including peritonitis-associated death, peritonitis-associated HD transfer, and recurrent/relapsing peritonitis were considered as treatment failure.

Long-term prognosis, including continued PD, transfer to permanent HD, or death, were assessed through follow-up. The time origin for follow-up was defined as the date of peritonitis onset. Patients who transferred to HD or underwent kidney transplantation were not treated as censoring events. All patients in the study were continuously followed for survival until death or the study end date (July 2025).

### 2.4. Statistical methods

Statistical analyses were performed using IBM SPSS Statistics 25.0, R language (version 4.3.3), and Zstats (version 1.0). To control for important confounding factors at baseline, 1:1 propensity score matching was applied. Normally distributed quantitative data were presented as mean ± standard deviation and compared between groups using the independent samples *t*-test. Continuous variables with non-normal distribution were expressed as median (interquartile range) and compared using the Mann-Whitney *U* test. Categorical variables were presented as frequency (percentage) and compared using the chi-square test or Fisher’s exact test. Receiver operating characteristic (ROC) curve analysis was used to evaluate the predictive value of each indicator for treatment failure in short-term prognosis, with the area under the curve as the primary measure. Based on interaction term testing by infection type (NF-GNB vs. F-GNB), univariate logistic regression analysis was conducted to assess the risk of treatment failure across different subgroups, calculating odds ratios with corresponding 95% confidence intervals, and forest plots were generated for visualization. For long-term prognosis analysis, Multivariate Cox regression was performed to explore potential predictors of mortality, Kaplan–Meier method was employed to plot survival curves, and the log-rank test was applied for between-group comparisons. All hypothesis tests in this study were two-sided, with *p* < 0.05 considered statistically significant.

## 3. Results

### 3.1. General information

**Figure 1.**
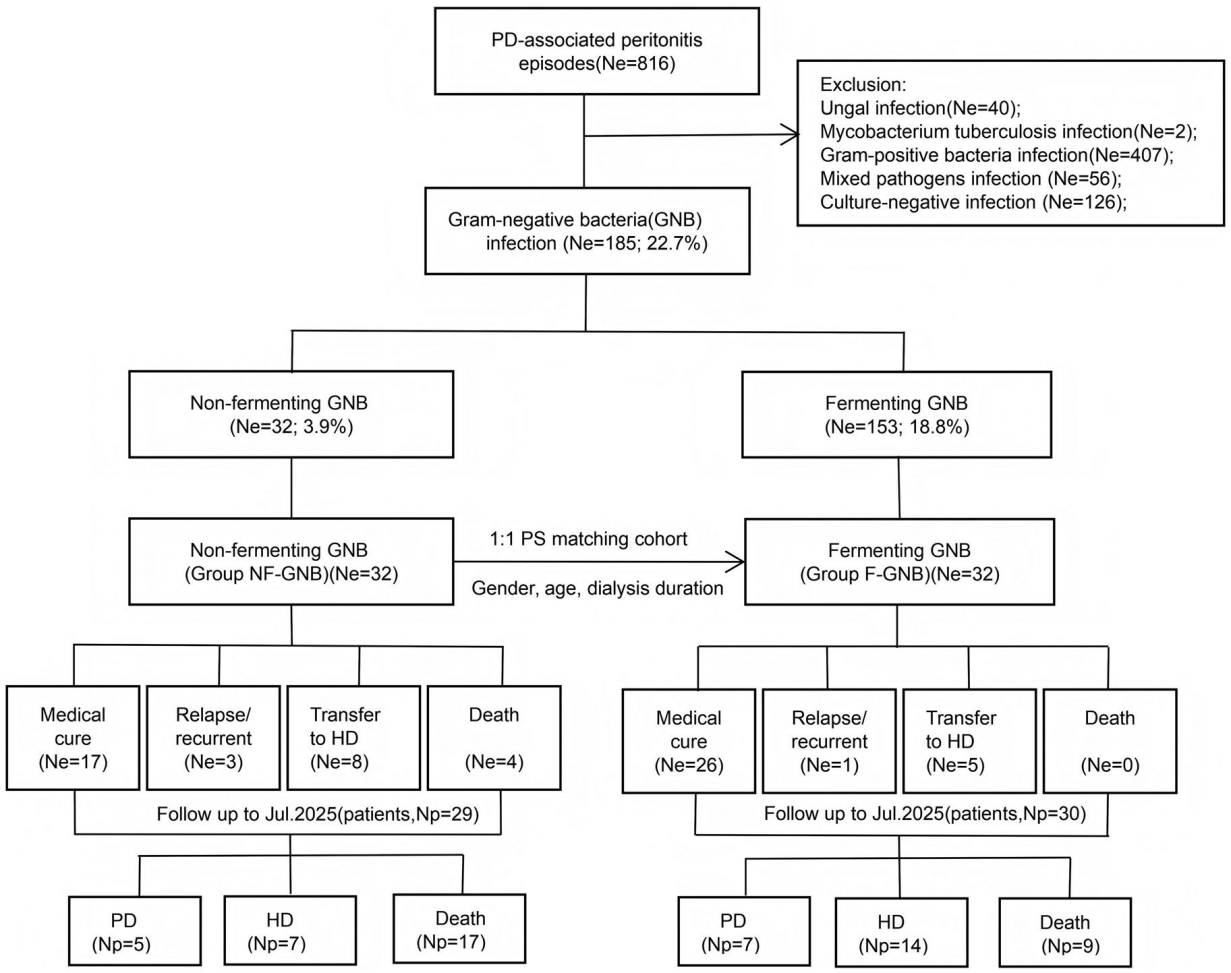
Patient flow chart.

From January 2010 to December 2024, a total of 816 PDAP episodes were recorded at our center. Among these, 40 episodes were attributed to fungal infections, 2 to *Mycobacterium tuberculosis* infection, 407 to GPB infections, 56 to mixed bacterial infections, 126 to culture-negative infections, and 185 to GNB infections. Among the GNB infection, NF-GNB infection occurred in 32 episodes involving 29 patients, constituting 3.9% of all PDAP episodes, and were classified into the NF-GNB group. F-GNB infection occurred in 153 episodes. Using 1:1 matching based on sex, age, and dialysis duration, 32 episodes of F-GNB infection (corresponding to 30 patients) were selected as the F-GNB group. The specific process is illustrated in Figure 1.

In the NF-GNB group, there were 12 episodes in males and 20 in females, with a mean age of 56.3 ± 16.7 years and a median dialysis duration of 52.0 months (IQR: 12.2–82.0). Among them, 24 episodes (75.0%) presented with their first episode of PDAP, while 8 episodes (25.0%) had a history of previous PDAP. Primary renal diseases included chronic glomerulonephritis (23 cases), diabetic nephropathy (7 cases), lupus nephritis (1 case), and obstructive nephropathy (1 case). Common comorbidities comprised hypertension (29 cases), coronary heart disease (13 cases), and diabetes mellitus (14 cases).

In the F-GNB group, there were 12 episodes in males and 20 in females, with a mean age of 56.7 ± 12.3 years and a median dialysis duration of 45.5 months (IQR: 28.4–80.7). Among them, 18 episodes (56.2%) presented with their first episode of PDAP, whereas 14 episodes (43.8%) had a history of previous PDAP. Primary renal diseases included chronic glomerulonephritis (28 cases), diabetic nephropathy (1 case), ANCA-associated nephritis (1 case), and polycystic kidney disease (2 cases). Common comorbidities included hypertension (22 cases), coronary heart disease (10 cases), and diabetes mellitus (2 cases).

### 3.2. Clinical symptoms and microbiological findings

In the NF-GNB group, the main clinical symptoms were cloudy dialysate (30 cases) and abdominal pain (29 cases), followed by fever (12 cases), diarrhea (4 cases), and nausea/vomiting (3 cases). Pathogenic testing of the peritoneal dialysate revealed that the predominant pathogens were *Pseudomonas* species (13 cases, 40.6%) and *Acinetobacter* species (11 cases, 34.4%). The remaining 8 cases (25.0%) were distributed as follows: Moraxella species (3 cases), *Sphingomonas paucimobilis*, *Stenotrophomonas maltophilia, Burkholderia cepacia, Brevundimonas diminuta,* and *Achromobacter* (1 case each). Among the 32 NF-GNB isolates, carbapenem resistance was observed in 6 (18.8%), with a carbapenem resistance rate of 27.3% for *Acinetobacter* species and 23.1% for *Pseudomonas* species.

In the F-GNB group, the main clinical symptoms were also cloudy dialysate (29 cases) and abdominal pain (28 cases), followed by fever (17 cases), diarrhea (2 cases), and nausea/vomiting (2 cases). Pathogenic testing of the peritoneal dialysate showed that *Escherichia* species were identified in 22 cases, *Klebsiella* species in 7 cases, *Pantoea* species in 1 case, and *Enterobacter cloacae* in 2 cases.

### 3.3. Comparison of characteristics and laboratory indicators between groups

A comparative analysis of baseline characteristics was conducted between patients in the NF-GNB group and the F-GNB group. For the matching variables (sex, age, and dialysis duration), there were no statistically significant differences between the two groups, indicating successful matching. The NF-GNB group had a higher proportion of patients with diabetes mellitus, a higher CCI score, and a lower body temperature at admission compared to the F-GNB group (all *p* < 0.05). No statistically significant differences were observed between the two groups in terms of coronary heart disease, hypertension, history of peritonitis, educational attainment, primary renal disease, *Kt/V*, BMI, heart rate, or blood pressure at admission.

Regarding laboratory indicators, the serum levels of NLR, PTH, albumin, and calcium in the NF-GNB group were significantly lower than those in the F-GNB group (all *p* < 0.05), no statistically significant differences were observed between the two groups in terms of blood WBC count, neutrophil count, lymphocyte count, hemoglobin, PCT, CRP, ferritin, HDL, LDL, LDH, ALP, potassium, or dialysate WBC count on day 1 and day 3. Detailed data are presented in Table 1.

**Table 1.** Comparison of baseline characteristics and laboratory tests between the two groups.

Variables	NF-GNB group	F-GNB group	*p*-Value
Age, years	56.3 ± 16.7	56.7 ± 12.3	0.952
Sex, Male/Female	12/20	12/20	1.000
Dialysis duration, months	52.0 (12.2, 82.0)	45.5 (28.4, 80.7)	0.648
Diabetes mellitus, *n* (%)	14 (43.8)	2 (6.3)	<0.001
Hypertension, *n* (%)	29 (90.6)	22 (68.8)	0.060
Coronary heart disease, *n* (%)	13 (40.6)	10 (31.3)	0.434
History of previous PDAP, *n* (%)	8 (25.0)	14 (43.8)	0.188
Charlson comorbidity index, *n* (%)			0.021
2 (low)	6 (18.8)	14 (43.8)	
3–4 (intermediate)	17 (53.1)	16 (50.0)	
5 (severe)	9 (28.1)	2 (6.3)	
Educational attainment, *n* (%)			0.405
Primary education	17 (53.1)	19 (59.4)	
Secondary education	14 (43.8)	10 (31.3)	
Tertiary education	1 (3.1)	3 (9.4)	
Primary renal disease, *n* (%)			0.103
Chronic nephritis	23 (71.9)	28 (87.5)	
Diabetic nephropathy	7 (21.9)	1 (3.1)	
Others	2 (6.3)	3 (9.4)	
*Kt/V*	2.17 ± 0.47	2.06 ± 0.38	0.347
BMI, kg/m^2^	22.8 ± 3.6	21.8 ± 3.1	0.227
Temperature, °C	37.3 ± 0.7	37.7 ± 0.8	0.049
Heart rate, bmp	90.7 ± 16.7	92.2 ± 19.5	0.742
Systolic pressure, mmHg	131.1 ± 30.9	131.6 ± 31.2	0.946
Diastolic pressure, mmHg	77.2 ± 16.9	78.1 ± 15.1	0.822
Blood WBC count,10^9^/L	8.6 ± 3.6	9.9 ± 3.4	0.160
Neutrophil count,10^9^/L	7.4 ± 3.4	8.7 ± 3.3	0.219
Lymphocyte count, 10^9^/L	0.8 (0.5, 1.0)	0.6 (0.4, 0.8)	0.082
NLR	10.3 (5.3, 13.2)	13.9 (8.9, 21.2)	0.027
Hemoglobin,g/L	101.8 ± 19.8	97.7 ± 15.6	0.358
CRP, mg/L	120.8 ± 73.3	138.1 ± 68.4	0.334
PCT	2.5 (0.8, 7.2)	6.4 (0.98, 23.8)	0.144
Albumin, g/L	27.0 ± 5.3	30.1 ± 3.6	0.008
HDL, mmol/L	1.08 ± 0.34	1.20 ± 0.33	0.189
LDL, mmol/L	1.96 ± 0.68	2.06 ± 0.67	0.543
ALP, U/L	87 (69, 104)	77 (53, 127)	0.339
LDH, U/L	206.6 ± 62.9	190.9 ± 53.3	0.296
Potassium, mmol/L	3.85 ± 0.78	4.08 ± 0.72	0.229
Calcium, mmol/L	2.21 ± 0.26	2.32 ± 0.19	0.049
Ferritin, ng/ml	269.9 (168.7, 411.7)	203.4 (106.2, 332.2)	0.163
PTH, pg/ml	131.0 (66.0, 222.0)	254.5 (139.5, 409.7)	0.004
Dialysate WBC count on day 1, /μL	2440.0 (1085.0, 6890.0)	2352.5 (857.5, 6125.0)	0.898
Dialysate WBC count on day 3, /μL	214.5 (61.3, 672.5)	159.0 (61.8, 571.8)	0.477

Abbreviations: NF-GNB: non-fermenting Gram-negative bacteria; F-GNB: fermenting Gram-negative bacteria; Kt/V: the urea clearance index; BMI: body mass index; WBC: white blood cell; NLR: neutrophil-to-lymphocyte ratio; CRP: C-reactive protein; PCT: procalcitonin; HDL: high-density lipoprotein; LDL: low-density lipoprotein; ALP: alkaline phosphatase; LDH: lactate dehydrogenase; PTH: parathyroid hormone.

### 3.4. Antibiotic therapy

Empirical antibiotic regimens in the NF-GNB group were mainly amikacin plus cefazolin (15 cases), amikacin plus vancomycin (5 cases), and third-generation cephalosporins (ceftriaxone, cefoperazone, or ceftizoxime) plus vancomycin (12 cases); in the F-GNB group, they were mainly amikacin plus cefazolin (12 cases), amikacin plus vancomycin (7 cases), and third-generation cephalosporins (ceftriaxone, cefoperazone, or ceftizoxime) plus vancomycin (13 cases). There was no statistically significant difference in the distribution of empirical antibiotic regimens between the two groups. After the culture results were obtained, either cefazolin or vancomycin was discontinued.

Based on the antimicrobial susceptibility testing results, 84.4% of patients had their antibiotic regimen adjusted or received a second antibiotic. The most commonly added second antibiotics included piperacillin-tazobactam, meropenem, and ciprofloxacin. The proportion of combination therapy was 75.0% (24/32) in the NF-GNB group, compared to 56.2% (18/32) in the F-GNB group, with no statistically significant difference between the two groups (*p* = 0.114). The median treatment duration was 18.5 days (IQR: 14–28) in the NF-GNB group and 16.5 days (IQR: 14–24.5) in the F-GNB group, with no statistically significant difference between the two groups (*p* = 0.898).

### 3.5. Short-term prognosis

In the NF-GNB group, 17 cases (53.1%) achieved medical cure, including 8 cases (72.7%, 8/11) in *Acinetobacter* species subgroup, 6 cases (46.2%, 6/13) in *Pseudomonas* species subgroup, and 3 cases (37.5%, 3/8) in other species subgroup, with no statistically significant differences among the subgroups (*p* = 0.272). The renmaining 15 cases were classified as treatment failure, including 3 cases of recurrent/relapsing infection, 8 cases of conversion to HD, and 4 cases of peritonitis-associated death.

In the F-GNB group, 26 cases (81.3%) achieved medical cure. The remaining 6 cases of tereatment failure comprised 1 case of relapse and 5 cases of transitions to HD (Figure 1). Comparative analysis showed that the medical cure rate in the NF-GNB group was significantly lower than that in the F-GNB group (*p* = 0.017).

#### 3.5.1. Subgroup analysis of treatment failure by infection type

**Figure 2.**
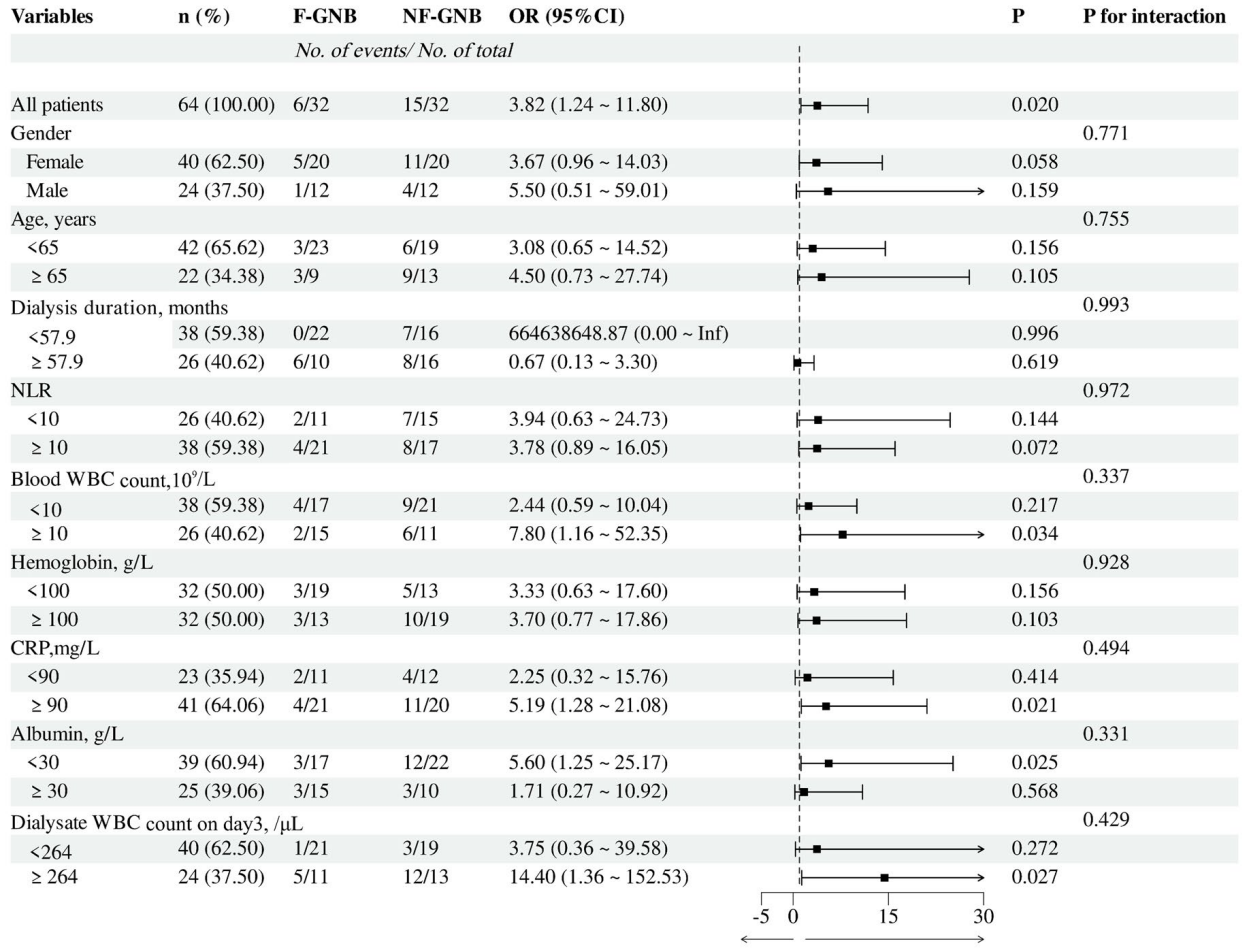
Subgroup analysis of treatment failure in patients with NF-GNB versus F-GNB infection.

To assess potential heterogeneity, a subgroup analysis was further conducted on patients with treatment failure, stratified by infection type (NF-GNB vs. F-GNB) (Figure 2). The analysis was layered according to the following variables: age (< 65 vs. ≥ 65 years), sex (Female vs. Male), hemoglobin (< 100 vs. ≥ 100 g/L), albumin (< 30 vs. ≥ 30 g/L), dialysis duration (< 57.9 vs. ≥ 57.9 months), blood WBC count (< 10 × 10^9^/L vs. ≥ 10 × 10^9^/L), CRP (< 90 vs. ≥ 90 mg/L), NLR (< 10 vs. ≥ 10), and dialysate WBC count on day 3 (< 264 vs. ≥ 264/μL). The results indicated that the risk of treatment failure in the NF-GNB group was 3.82 times that in the F-GNB group (OR = 3.82, 95% CI: 1.24–12.8, *p* = 0.020). All P-values for interaction tests were > 0.05, suggesting no significant interaction effect overall. However, in certain subgroups, patients with NF-GNB infection had a significantly higher risk of treatment failure, including: CRP ≥ 90 mg/L (OR = 5.19, 95% CI: 1.28–21.08, *p* = 0.021), blood WBC count ≥ 10 × 10^9^/L (OR = 7.80, 95% CI: 1.16–52.35, *p* = 0.034), albumin < 30 g/L (OR = 5.60, 95% CI: 1.25–25.17, *p* = 0.025), and dialysate WBC count on day 3 ≥ 264/μL (OR = 14.40, 95% CI: 1.36–152.53, *p* = 0.027). This suggests that among patients with NF-GNB infection, markedly elevated inflammatory markers (including high blood WBC count, high CRP, and elevated dialysate WBC count on day 3) or hypoalbuminemia may further increase the risk of treatment failure, highlighting the need for close monitoring and targeted intervention in this patient population.

#### 3.5.2. ROC curve analysis of predictors for treatment failure in the NF-GNB group

**Figure 3.**
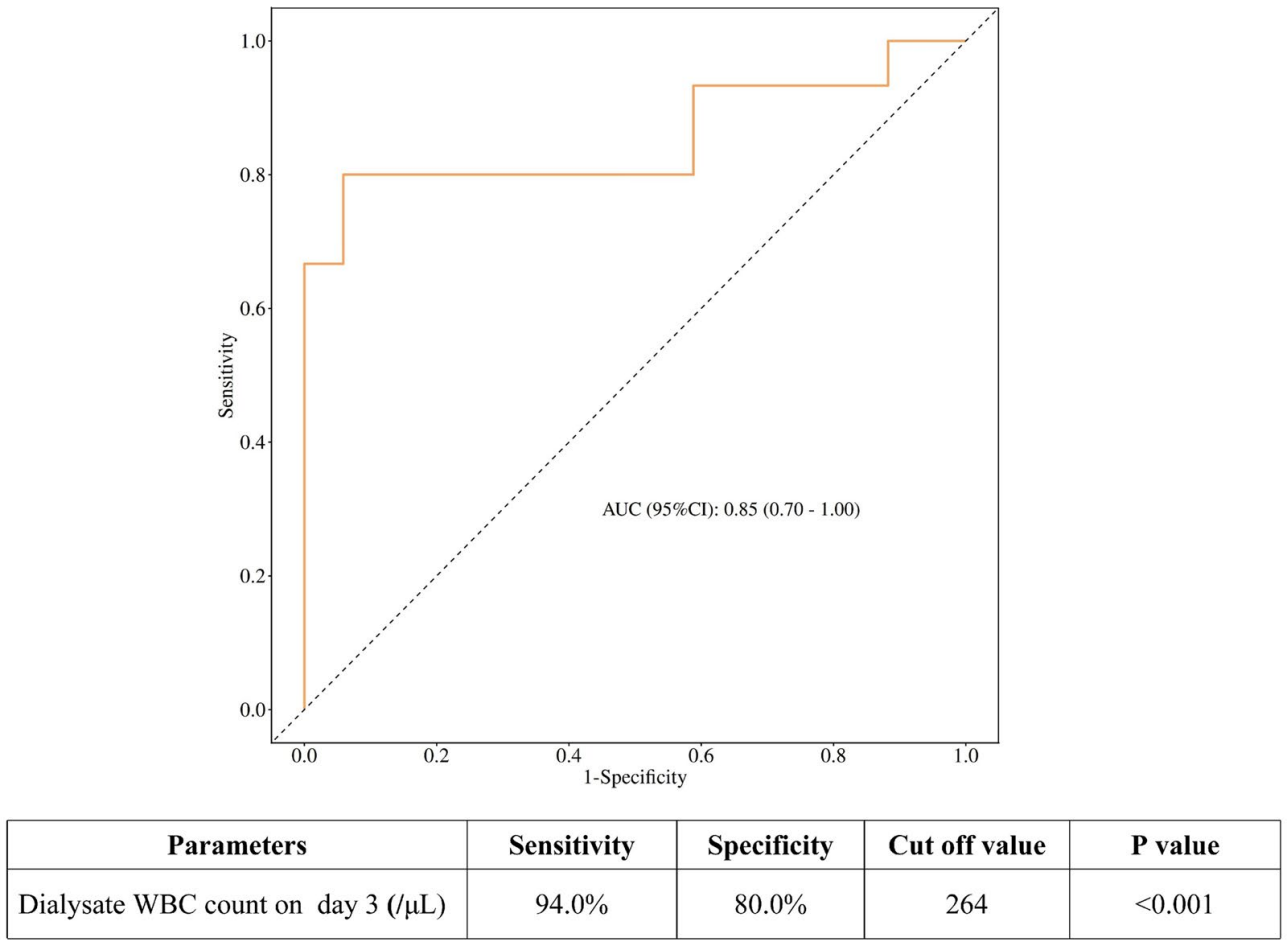
ROC curve analysis of dialysate WBC count on day 3 for treatment failure.

To explore predictors of treatment failure in the NF-GNB group, ROC curve analysis was performed on all continuous variables. The results showed that the dialysate WBC count on day 3 had predictive value for treatment failure (*p* < 0.001) (Figure 3), with an AUC of 0.85. The exploratory analysis suggested a cutoff of 264/μL, with a sensitivity of 94.0% and a specificity of 80.0%.

### 3.6. Long-term prognosis

All patients were followed up until the study endpoint or death, the follow-up duration was calculated from the date of peritonitis onset and ranged from 0.3 to 181.7 months. The median follow-up duration was 33.2 months (IQR: 10.1–91.8) in the NF-GNB group and 59.6 months (IQR: 34.1–95.2) in the F-GNB group, with no statistically significant difference between the two groups (*p* = 0.087).

By the last follow-up, 17 deaths (58.6%, 17/29) had occurred in the NF-GNB group: 7 patients (70.0%, 7/10) in the *Pseudomonas* species subgroup, 6 patients (54.5%, 6/11) in the *Acinetobacter* species subgroup, and 4 patients (50.0%, 4/8) in the other species subgroup. No significant difference in mortality was observed among these pathogen subgroups (*p* = 0.712). In the F-GNB group, 9 deaths (30.0%, 9/30) were recorded. Overall mortality was significantly higher in the NF-GNB group than in the F-GNB group (*p* = 0.027). Among the survivors, 5 patients in the NF-GNB group and 7 in the F-GNB group remained on PD (*p* = 0.750), while 7 patients in the NF-GNB group and 14 patients in the F-GNB group had switched to permanent HD (*p* = 0.071). Detailed data are presented in Figure 1.

To explore risk factors associated with long-term mortality, we performed a Cox proportional hazards regression analysis. Univariate analysis revealed that the following factors might be associated with an increased risk of death: NF-GNB infection (HR = 2.31, 95% CI: 1.03–5.20, *p* = 0.043), older age (HR = 1.04, 95% CI: 1.01–1.07, *p* = 0.028), *Kt/V* < 1.7 (HR = 4.43, 95% CI: 1.71–11.43, *p* = 0.002), dialysate WBC count on day 3 ≥ 264/μL (HR = 2.63, 95% CI: 1.20–5.75, *p* = 0.015), and hypokalemia (HR = 2.32, 95% CI: 1.06–5.11, *p* = 0.036). No significant association with mortality was found for sex, CCI, dialysis duration, educational attainment, previous PDAP, diabetes mellitus, hypertension, coronary heart disease, serum albumin, hemoglobin, CRP, BMI, PCT, ferritin, ALP, LDH, PTH, HDL, LDL, NLR, calcium, or vital signs on admission (body temperature, blood pressure, and heart rate).

Based on the univariate analysis results, we further constructed a multivariate Cox proportional hazards regression model. First, building upon the model containing only the NF-GNB infection variable (Model 1), we adjusted for covariates including sex, age, dialysis duration, previous PDAP, CCI and hypertension to establish Model 2. The results indicated that NF-GNB infection remained significantly associated with the risk of all-cause mortality. Subsequently, on the basis of Model 2, we further incorporated the dialysate WBC count on day 3, *Kt/V*, and hypokalemia to build the final model (Model 3). Analysis of Model 3 showed that NF-GNB infection (HR = 3.40, 95% CI: 1.05–11.00, *p* = 0.041) and lower *Kt/V* value (HR = 12.54, 95% CI: 2.32–67.94, *p* = 0.003) might be associated with the risk of death. In contrast, age, dialysate WBC count on day 3, and hypokalemia showed no significant association. Detailed data are presented in Table 2.

**Table 2.** Risk factors associated with death in cox regression models.

Variables	Model 1		Model 2		Model 3	
	HR (95%CI)	*P*	HR (95%CI)	*P*	HR (95%CI)	*P*
Group (NF-GNB vs. F-GNB)	2.31 (1.03–5.20)	0.043	2.83 (1.11–7.24)	0.030	3.40 (1.05 ∼ 11.00)	0.041
Sex (Male vs. Female)			1.19 (0.44–3.20)	0.732	0.53 (0.11–2.46)	0.415
Age, years			1.04 (1.01–1.07)	0.023	1.02 (0.98–1.06)	0.269
Dialysis duration, months			1.00 (0.99–1.01)	0.922	1.00 (0.99–1.01)	0.985
Previous PDAP (Yes vs. No)			0.90 (0.29–2.78)	0.859	0.90 (0.25–3.27)	0.868
CCI						
Low			1.00 (Reference)		1.00 (Reference)	
Intermediate			1.96 (0.72–5.34)	0.188	1.54 (0.49–4.82)	0.457
Severe			1.46 (0.37–5.77)	0.587	1.09 (0.13–9.15)	0.939
Hypertension (Yes vs. No)			0.42 (0.14–1.25)	0.120	0.47 (0.14–1.64)	0.238
*Kt/V* (< 1.7 vs. ≥ 1.7)					12.54 (2.32–67.94)	0.003
Dialysate WBC count onday 3 (≥ 264 /μL vs. < 264 /μL)					2.48 (0.78–7.85)	0.122
Hypokalemia (Yes vs. No)					1.78 (0.50–6.33)	0.374
HR: Hazard Ratio, CI: Confidence Interval						
Model 1: Crude						
Model 2: Adjust: Model 1 + Sex, Age, Dialysis duration, Previous PDAP, CCI, Hypertension						
Model 3: Adjust: Model 2 + *Kt/V*, Dialysate WBC count on day 3, Hypokalemia						

Abbreviations: HR: Hazard Ratio; CI: confidence interval; NF-GNB: non-fermenting Gram-negative bacteria; F-GNB: fermenting Gram-negative bacteria; CCI: Charlson comorbidity index; Kt/V: the urea clearance index.

**Figure 4.**
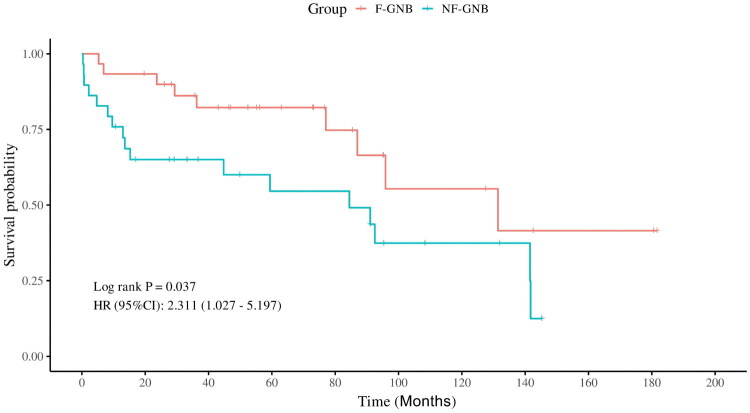
Kaplan–Meier survival analysis in two groups.

We compared the survival rates of the two groups at different time points. The survival rates in the NF-GNB group at 1 year, 3 years, 5 years, 10 years, and beyond 10 years after the onset of peritonitis were 75.9%, 58.3%, 45.5%, 21.1%, and 10.5%, respectively. In the F-GNB group, the corresponding survival rates were 93.3%, 85.2%, 75.0%, 38.5%, and 30.8%, with the P values for the intergroup comparisons were 0.080, 0.032, 0.051, 0.427, and 0.194, respectively. The NF-GNB group exhibited consistently lower survival rates than that of the F-GNB group throughout the follow-up period, with significant differences observed mainly within 3 years after peritonitis. Kaplan-Meier survival analysis further confirmed that the overall survival rate was significantly lower in the NF-GNB group than in the F-GNB group (log-rank *χ*^2^ = 4.343, *p* = 0.037) (Figure 4).

## 4. Discussion

This study reviewed and analyzed cases of PDAP caused by NF-GNB at our center, where the incidence is relatively low. Overall, GNB infection accounted for 22.7% (185/816) of all PDAP episodes during the study period, with only 3.9% (32/816) attributed to NF-GNB. Among NF-GNB infection, *Pseudomonas* and *Acinetobacter* species were the predominant pathogens, accounting for 40.6% and 34.4% of cases, respectively. Dos Santos et al. [9] reported a comparable proportion of GNB (26.7%) in PDAP but a higher proportion of NF-GNB (9.5%) than in our study. In their study, *Pseudomonas* and *Acinetobacter* species were also the most common pathogens, comprising 51.6% and 32.2% of NF-GNB infection, respectively.

Although the incidence of NF-GNB PDAP is low, the overall prognosis is poor, and findings vary among studies. In the present study, the overall cure rate was 53.1%, with subgroup rates of 46.2% for *Pseudomonas* species, 72.7% for *Acinetobacter* species, and 37.5% for other species. Due to the small sample size, the inter-subgroup difference did not reach statistical significance; however, the cure rate for *Pseudomonas* species showed a trend toward being lower than that for *Acinetobacter* species. These findings are generally consistent with those reported by Li et al. [13], who documented cure rates of 45% for *Pseudomonas* species and 77% for *Acinetobacter* species in PDAP cases from Hong Kong between 2000 and 2015. In contrast, Dos Santos et al. [9] reported lower cure rates in 62 cases of NF-GNB PDAP from Brazil between 1997 and 2015, with an overall cure rate of only 32.2% (31.2% for *Pseudomonas* species, 40.0% for *Acinetobacter* species, and 22.2% for other species, respectively), they suggested that the poor prognosis of NF-GNB infection might be associated with the synergistic effect of biofilm formation and virulence factors [9,10]. Furthermore, several studies focusing on specific pathogens have reported varying outcomes: Dong et al. [14] described a complete cure rate of 40.4% in 57 cases of *Pseudomonas* peritonitis from Southern China between 2008 and 2022; Davenport et al. [15] and Szeto et al. [16] reported even lower cure rates for *Pseudomonas* PDAP, at 21.4% and 22.1%, respectively. Regarding *Acinetobacter* infections, Htay et al. [17] reported a cure rate of 74% for *Acinetobacter* PDAP in Australia between 2004 and 2014, which was associated with approximately 300% higher odds of cure compared with *Pseudomonas* PDAP.

In this study, compared to F-GNB group, the NF-GNB group had a higher proportion of comorbid diabetes, higher CCI scores, and relatively lower body temperature at admission, as well as lower serum albumin, PTH, calcium, and NLR.

Diabetes mellitus is a common metabolic disorder in middle-aged and elderly populations. In the study by Troidle et al. [7], a higher proportion of patients with diabetes developed Gram-negative peritonitis compared to Gram-positive peritonitis. Chronic hyperglycemia not only provides an ideal environment for pathogen growth by enhancing bacterial proliferation and virulence, but also impairs immune function—manifested specifically as reduced leukocyte adhesion and enhanced chemotactic responses, and may furthermore contribute to the development of antibiotic resistance [18,19]. This may partially explain the increased risk of opportunistic infections among diabetic patients in the NF-GNB group. Furthermore, as diabetes is one of the conditions assessed in the CCI, it also contributed to the elevated CCI scores observed in the NF-GNB group.

In this study, the proportion of patients with fever at admission and the mean body temperature were lower in the NF-GNB group than in the F-GNB group. Fever during the early phase of inflammation is generally considered a beneficial adaptive response, indicating that the body is activating defense mechanisms against pathogens. However, in cases of severe infection or when the body’s energy reserves are insufficient, body temperature may fail to rise or may even decrease. Under such circumstances, mortality has been shown to be inversely correlated with body temperature [20–22]. Against the background of markedly elevated systemic inflammatory markers, the absence of a febrile response may suggest an inadequate host response to severe infection. This phenomenon was more pronounced in the NF-GNB group.

Serum albumin is a key indicator of nutritional status, it also plays a role in immune regulation and contributes to maintaining various homeostatic functions [23]. Furthermore, many metabolites known to influence the immune system circulate primarily in a form bound to albumin [24]. Hypoalbuminemia has been confirmed by multiple studies as an important risk factor for developing PDAP [25–27]. In the present study, the mean albumin level was lower in the NF-GNB group than in the F-GNB group. Subgroup analysis further revealed that among patients with serum albumin below 30 g/L, the risk of treatment failure in the NF-GNB group was 5.60 times higher than that in the F-GNB group. These findings highlight the clinical importance of monitoring and improving albumin levels in peritoneal dialysis patients.

PTH is not only a core hormone regulating calcium and phosphorus metabolism but is also involved in immune modulation. Reduced PTH levels often indicate a state of malnutrition and may increase susceptibility to infections in dialysis patients. Hong et al. [28] found that PTH is an independent predictor of infection-related mortality in incident dialysis patients. Furthermore, blood calcium levels are regulated by PTH and also bind to albumin to form bound calcium. In the present study, the NF-GNB group exhibited lower serum calcium levels, consistent with their lower PTH and albumin levels compared to the F-GNB group.

The NLR is a commonly used marker for assessing systemic inflammation and immune dysregulation, with a typical reference range of 0.78–3.53 in healthy adults [29]. Yu et al. [1] from our center previously reported a median NLR of 7.3 in 648 PDAP patients and identified NLR as an independent predictor of peritonitis-related technique failure. In the present study, the median NLR values were 10.3 in the NF-GNB group and 13.9 in the F-GNB group, both markedly higher than the previously reported median for the overall PDAP patients at our center. However, although the NLR was higher in the F-GNB group than in the NF-GNB group, this marker did not demonstrate significant predictive value for short- or long-term prognosis in patients with PDAP, which may be attributable to the selected study population or the relatively small sample size.

Regarding short-term prognosis, subgroup analysis showed that the NF-GNB group had a higher risk of treatment failure than the F-GNB group. To explore potential predictors of treatment failure in the NF-GNB group, ROC curve analysis was performed on continuous variables. The results suggested an exploratory cutoff of dialysate WBC count on day 3 ≥ 264/μL for predicting treatment failure. Elevated dialysate WBC count on day 3 directly reflects poor intraperitoneal inflammation control, suggesting that initial antibiotic therapy may be ineffective or that the infection is more severe.

The 2022 ISPD peritonitis guidelines define refractory peritonitis as dialysate WBC count on day 5 > 100/μL and consider it an early warning indicator of treatment failure [3,30]. The present study advanced the assessment time point to day 3 and, within a small NF-GNB subgroup, identified an exploratory cutoff of 264/μL. This cutoff yielded an AUC of 0.85, a sensitivity of 94.0%, and a specificity of 80.0% for predicting treatment failure. Based on this exploratory finding, patients with NF-GNB infection could potentially be stratified on day 3 into a high-risk group (≥264/μL) and a low-risk group (<264/μL). Those in the high-risk group may require more intensive interventions to reduce the risk of treatment failure, whereas those in the low-risk group could continue standard therapy to avoid overtreatment. It should be emphasized that this cutoff was derived from a small subgroup and has not been externally validated; therefore, it should be interpreted with caution. Similarly, Chow et al. [31] identified a dialysate WBC count on day 3 ≥ 1090/μL as a predictor of PDAP treatment failure, with a sensitivity of 75% and a specificity of 74%. In addition, our study found that a dialysate WBC count on day 3 ≥ 264/μL was a predictor of long-term mortality in univariate Cox analysis but did not retain independent predictive value in the multivariable analysis. Notably, because of the limited sample size in this study, future larger scale studies are needed to further validate this cutoff and its association with long-term prognosis.

Regarding long-term prognosis in our study, the mortality was significantly higher in the NF-GNB group than in the F-GNB group. Adjusting for confounders using a multivariate Cox regression model, the results showed that NF-GNB infection and a lower *Kt/V* value might be associated with an increased risk of death. *Kt/V* is a key metric for assessing small-solute clearance and dialysis adequacy [32]. The 2006 ISPD guidelines [33] first explicitly recommended that the total weekly *Kt/V* should be ≥ 1.7 in patients on CAPD. In PD patients, a *Kt/V* < 1.7 has been confirmed as an independent predictor of mortality [34–36]. Our study further demonstrated that a *Kt/V* < 1.7 is also an independent predictor of death in PDAP patients. Therefore, dialysis adequacy is closely related to patient prognosis. Clinicians should pay close attention to *Kt/V* levels during routine follow-up and promptly adjust the dialysis prescription for patients who do not achieve the target *Kt/V*.

In conclusion, this retrospective, single-center exploratory analysis of NF-GNB PDAP suggests that patients with NF-GNB infection may experience poorer short- and long-term clinical prognosis than those with F-GNB infection. Clinicians should maintain a high index of suspicion for such infections, as early identification of high-risk patients and implementation of intensified treatment strategies may be important for improving survival. However, given the exploratory nature of this single-center study, these findings should be interpreted cautiously and require external validation through future multicenter, prospective research.

## Data Availability

The datasets utilized and analyzed in this study can be obtained from the corresponding author upon reasonable request.
